# Attention-Deficit/Hyperactivity Disorder, Its Pharmacotherapy, and Adrenal Gland Dysfunction: A Nationwide Population-Based Study in Taiwan

**DOI:** 10.3390/ijerph17103709

**Published:** 2020-05-25

**Authors:** Pin-Han Peng, Meng-Yun Tsai, Sheng-Yu Lee, Po-Cheng Liao, Yu-Chiau Shyu, Liang-Jen Wang

**Affiliations:** 1 Department of Psychiatry, Kaohsiung Chang Gung Memorial Hospital and Chang Gung University College of Medicine, Kaohsiung 833, Taiwan; pengpinhan@gmail.com; 2 Department of Internal Medicine, Kaohsiung Chang Gung Memorial Hospital and Chang Gung University College of Medicine, Kaohsiung 833, Taiwan; ck940176@gmail.com; 3 Department of Psychiatry, Kaohsiung Veterans General Hospital, Kaohsiung 813, Taiwan; shirleylee.ncku@gmail.com; 4 Department of Psychiatry, College of Medicine, Graduate Institute of Medicine, School of Medicine, Kaohsiung Medical University, Kaohsiung 807, Taiwan; 5 Community Medicine Research Center, Keelung Chang Gung Memorial Hospital, Keelung 204, Taiwan; henryshome@gmail.com; 6 Department of Nursing, Chang Gung University of Science and Technology, Taoyuan City 333, Taiwan; 7 Institute of Molecular Biology, Academia Sinica, Taipei 115, Taiwan; 8 Department of Child and Adolescent Psychiatry, Kaohsiung Chang Gung Memorial Hospital and Chang Gung University College of Medicine, Kaohsiung 833, Taiwan

**Keywords:** ADHD, comorbidity, adrenal gland dysfunction, epidemiology, pharmacotherapy

## Abstract

This study aims to examine the co-occurrence rate of attention deficit hyperactivity disorder (ADHD) and adrenal gland disorders, as well as whether pharmacotherapy may affect ADHD patients’ risk of developing adrenal gland disorder. One group of patients newly diagnosed with ADHD (*n* = 75,247) and one group of age- and gender-matching controls (*n* = 75,247) were chosen from Taiwan′s National Health Insurance database during the period of January 1999 to December 2011. Both patients and controls were monitored through December 31, 2011, in order to identify the occurrence of adrenal gland disorders (ICD-9-CM code 255.X). We also explored the potential effect of methylphenidate (MPH) and atomoxetine (ATX) treatments on the risk of developing adrenal gland disorders. We found that ADHD patients showed a significantly increased probability of developing an adrenal gland disorder compared to the control group (0.2% of ADHD vs. 0.1% of controls). However, neither MPH nor ATX treatment significantly influenced the patients’ risk of developing adrenal gland dysfunction. We propose that patients with ADHD had greater comorbid rates with adrenal gland dysfunction than the control subjects. Nevertheless, undergoing treatment with MPH or ATX did not significantly influence the risk of developing adrenal gland dysfunction among ADHD patients.

## 1. Introduction

Attention deficit hyperactivity disorder (ADHD) is a neuropsychiatric disorder that commonly occurs in children and adolescents, affecting about 7% of all school-age children worldwide [1], with a significant sex disparity in the prevalence (14% in boys and 6.3% in girls [2]. The core symptoms of ADHD are hyperactivity/impulsivity and inattention [3]. Although the pathogenesis of ADHD is not well known, a genetic imbalance of catecholamine metabolism in the cerebral cortex appears to play a major role, as illustrated by structural and functional brain imaging, animal studies, and the response to drugs with noradrenergic activity [4]. The glucocorticoid hormone cortisol, a main product of the hypothalamic-pituitary-adrenal (HPA) axis, is among the most frequently employed biomarkers in psychobiological research for the following reasons [5]; abnormal hypothalamic-pituitary-adrenal (HPA) axis function has been associated with the pathogenesis of ADHD. Cortisol levels are responsive to social and psychological stress [6,7]. Furthermore, cortisol has been found to play an important role in daily cognitive and behavioral functioning [8], and is also involved in the etiology of various mental and physical health outcomes [9].

Prior studies on children with ADHD that examined overall diurnal cortisol levels and rhythm have shown significantly lower basal cortisol concentrations in the morning [10,11,12] and a significantly lower incidence of typical diurnal variation [13]. A recent meta-analysis of case-control studies focusing on baseline cortisol showed a modest but significant effect (d = −0.31) of ADHD, showing lower levels in ADHD compared to control subjects [14]. A lower level of cortisol in children, also known as adrenal insufficiency, is defined by the impaired synthesis and release of adrenocortical hormones. Adrenal insufficiency can be classified into primary and secondary (or central), based on the mechanism. Primary adrenal insufficiency is caused by diseases intrinsic to the adrenal cortex, while secondary/central adrenal insufficiency results from impaired production of the adrenocorticotropic hormone (ACTH) [15]. Regardless of type, they both cause a lower level of cortisol, a condition also found in children with ADHD.

The management of ADHD includes behavioral therapy and medication, with methylphenidate (MPH), a psycho-stimulant, being the most commonly used drug. The dual mechanism of MPH consists of inhibiting the re-uptake of both dopamine and norepinephrine, leading to treatment effects for not only behavioral but also cognitive aspects [3]. Previous studies have indicated that MPH administration appears to influence cortisol levels, a label of functioning of the HPA axis, in ADHD patients [16,17,18]. The influence of MPH on the HPA and hypothalamic–pituitary–gonadal (HPG) axes may be through direct impact of the catecholamine system, or through its indirect influence on decreasing the impact of environmental stress on the HPA axis and HPG activity. Meanwhile, atomoxetine (ATX) is a selective norepinephrine re-uptake inhibitor that is classified as a nonstimulant [19] and is a second-line medication in the management of ADHD in children who are unresponsive to stimulants. Some researchers have found that children taking ATX had higher cortisol levels than unmedicated children, which may due to the noradrenaline augmenting HPA axis function increasing the corticotrophin releasing hormone (CRF) at the paraventricular nucleus of the hypothalamus [11,20]. However, whether MPH or ATX affects ADHD patients’ risk of developing adrenal gland disorder remains unclear.

The association and comorbidity of ADHD and adrenal gland disorders have yet to be fully explored. Therefore, we conducted this nationwide population-based analysis to explain the co-occurrence rate of ADHD and adrenal gland disorders. We also examined whether certain pharmacotherapy may affect the risk of developing adrenal gland disorder in ADHD patients.

## 2. Materials and Methods

### 2.1. Data Source

The institutional review board of Chang Gung Memorial Hospital has already approved this study, whose data came from the ambulatory claims database of the National Health Insurance Research Database of Taiwan (NHIRD-TW). First implemented in 1995, Taiwan’s National Health Insurance (NHI) program is a mandatory universal health insurance program, for which the NHI Bureau is the sole payer for health care services. Since the launch of the program, the NHI Bureau has contracted 93% of all healthcare providers in Taiwan, and at least 96% of the insured have used healthcare services once or more through those contracted hospitals and clinics. It is required that contracted medical institutions must electronically submit claim documents related to medical expenses every month. The reliability of NHIRD diagnostic codes has been proven in a previous study [21].

### 2.2. Ethical Approval

The protocol for this study conformed to the Helsinki Declaration, and was approved by the Institutional Review Board (IRB) of Chang Gung Memorial Hospital (No. 102-1297B). 

Patient records/information was anonymized and de-identified prior to analysis, and the need for written informed consent was waived by the IRB.

### 2.3. Selection of ADHD Patients

We recruited all patients newly diagnosed with ADHD between January 1999 and December 2011 with records found in the NHIRD-TW (*n* = 146,063). To reduce potential misdiagnosis, we defined ADHD as at least two NHI claim records with the International Classification of Diseases, 9th Edition, Clinical Modifications (ICD-9-CM) code 314.X. Moreover, we excluded the patients (*n* = 70,816) born after 31 December 1999, the reason of which will be explained later. After excluding these patients, our data set included 75,247 patients in the ADHD group. We defined the index date as the date when ADHD was first diagnosed and followed these patients’ medical records in the NHIRD-TW from the index date until 31 December 2011.

### 2.4. Selection of the Control Group

We selected the control group from another subset cohort of the NHIRD-TW, the Longitudinal Health Insurance Database 2000 (LHID2000), which is composed of the original claim data for one million beneficiaries randomly sampled from the NHIRD-TW’s 2000 Registry of Beneficiaries. The control subjects had no ADHD diagnosis between 1 January 1996, and 31 December 2011. In this study, we used the propensity score matching technique to create a matching control with a ratio of 1:1 for the ADHD group. Propensity scores were determined by multivariate logistic regression analysis, where gender and birth year were confounding covariates [22,23]. We determined an index date of each matched control, which was established as the ADHD diagnosis date from its matching ADHD cases, and then tracked all the 75,247 controls from this entry date until the end of the study period (31 December 2011).

### 2.5. Comorbidities and Outcomes

We further identified neurodevelopmental disorders that are commonly comorbid with ADHD, including oppositional defiant disorder (ODD) (ICD-9-CM code 313.81), conduct disorder (ICD-9-CM code 312.X), autism spectrum disorder (ASD) (ICD-9-CM code 299.X), tic disorder (ICD-9-CM code 307.2X), and intellectual disability (ICD-9-CM code 317 to 319).

For this study, we defined adrenal gland dysfunction as having a diagnosis of adrenal glands (ICD-9-CM code 255.X) and determined the diagnosis and diagnosis date of adrenal gland dysfunction based on insurance status and outpatient and inpatient claim databases. Adrenal gland disorder (ICD-9-CM 255.X) was classified into Cushing’s syndrome (ICD-9-CM 255.0), hyperaldosteronism (ICD-9-CM 255.1), adrenal genital disorders (ICD-9-CM 255.2), other corticoadrenal overactivity (ICD-9-CM 255.3), corticoadrenal insufficiency (ICD-9-CM 255.4), other adrenal hypofunction (ICD-9-CM 255.5), medulloadrenal hyperfunction (ICD-9-CM 255.6), other specified disorders of adrenal glands (ICD-9-CM 255.8), or an unspecified disorder of the adrenal glands (ICD-9-CM 255.9).

### 2.6. Definition of Pharmacotherapy

Medications were confirmed using the Anatomical Therapeutic Chemical classification system [24]. Methylphenidate (MPH) and atomoxetine (ATX) are two medications approved by the NHI Bureau for treating ADHD in Taiwan. Patients who had received medications were defined as those who had had any prescription record of MPH or ATX in an ambulatory care, pharmacy, or hospital care claim. To explore the potential impact of pharmacotherapy on developing adrenal gland dysfunction in ADHD patients, we tracked their medical records in the NHIRD-TW until 31 December 2011 or the development of adrenal gland dysfunction, whichever came first. We also recorded the duration and daily dose of MPH or ATX in use and converted the daily dose into the defined daily dose (DDD) determined by WHOCC Drug Statistics Methodology.

### 2.7. Statistical Analysis

We used the Statistical Package for the Social Sciences (SPSS) Version 16.0 (SPSS Inc., Chicago, IL, USA) to perform all the statistical analyses in this study. We considered a two-tailed value of *p* < 0.05 statistically significant.

We adopted the chi-square (χ^2^) test or *t*-test to compare characteristics between the ADHD group and the control group. Furthermore, we used a Cox regression model to examine the potential influence of ADHD on the comorbidity of adrenal gland dysfunction. A previous study reported that primary and secondary adrenal insufficiency are more frequently in women than in men [15], and the age at diagnosis peaks in the adulthood. Therefore, we recruited age of ADHD diagnosis, sex, and comorbidities as potential confounding factors in the statistical model. The survival analysis time function was calculated as the number of days from the initial observation until 31 December 2011 (end of follow-up). We also calculated the adjusted hazard ratio (aHR) and 95% confidence interval (CI).

We then utilized the multivariate logistic regression model to estimate the potential influence of pharmacotherapy on the risk of developing adrenal gland dysfunction, controlling for the effects of age, gender, and psychiatric comorbidities. We established three models (MPH only, ATX only, and MPH + ATX) to test whether MPH and ATX exerted differential effects on adrenal gland dysfunction. The multivariate logistic regression model was also used to estimate the potential effects of duration of MPH/ATX in use and the daily doses on the risk of developing disorders of adrenal glands. We calculated both the adjusted odds ratio (aOR) and the 95% confidence interval (CI).

### 2.8. Results

Table 1 shows the characteristics of the ADHD group (mean age: 9.8 years, 79.4% male) and the control group (mean age: 10.2 years, 69.1% male). The ADHD group had a younger age and higher proportion of males compared to the control group. Furthermore, the ADHD group had higher comorbidity rates of ODD (5.8%), conduct disorder (6.1%), tic disorders (6.5%), ASD (8.7%), and intellectual disability (14.3%). During the study period, 71.0% and 4.2% of ADHD patients received MPH and ATX treatment, respectively. Compared to the control group, the ADHD group had higher comorbidity rates of adrenal gland dysfunction (0.2% of ADHD vs. 0.1% of controls). The ages at which a patient was diagnosed with adrenal gland dysfunction did not differ significantly between the groups. For the specific diagnosis of adrenal gland dysfunction, the ADHD group had higher comorbidity rates of Cushing′s syndrome, adrenogenital disorders, corticoadrenal insufficiency, and unspecified disorder of adrenal glands than the control group (Supplementary Table S1).

**Figure 1:**
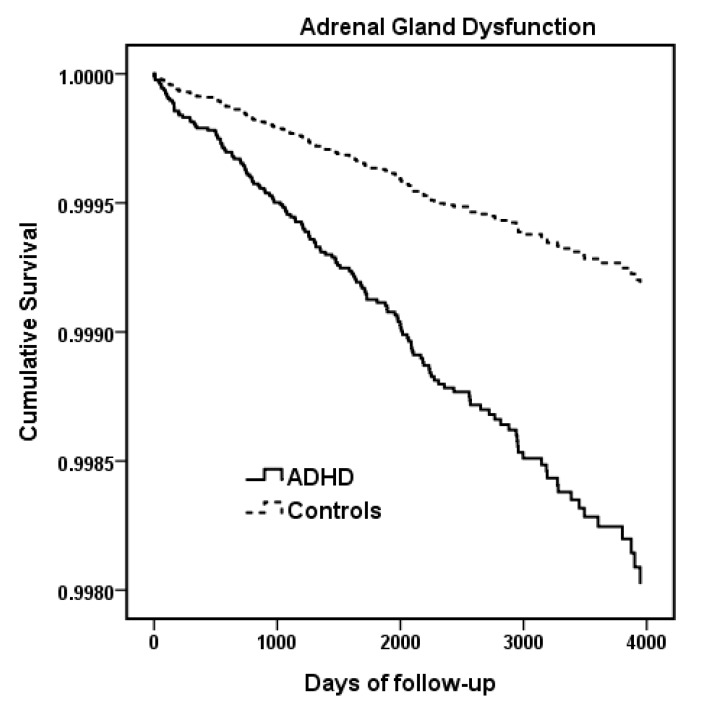
Survival function expressed by Cox regression of adrenal gland disorders among the ADHD and control groups.

The Cox regression models are shown in Table 2. The unadjusted model demonstrates that ADHD (aHR, 2.47; 95% CI, 1.73–3.52), an older age (aHR, 1.14; 95% CI, 1.10–1.18), comorbidities of conduct disorder (aHR, 2.07; 95% CI, 1.09–3.93), ASD (aHR, 1.96; 95% CI, 1.15–3.35), and intellectual disability (aHR, 1.79; 95% CI, 1.13–2.81) were all risk factors for an adrenal gland dysfunction diagnosis. The adjusted model also demonstrates that ADHD patients were more likely to be diagnosed with adrenal gland dysfunction (aHR, 2.40; 95% CI, 1.64–3.50) than the control subjects. The survival curve that was estimated using the Cox regression model is presented in Figure 1. Both an older recruitment age (aHR, 1.15; 95% CI, 1.11–1.19) and being female (aHR, 1.67; 95% CI, 1.18–2.38) were associated with a greater risk of adrenal gland dysfunction.

We used the multivariate logistic regression models to examine the potential influence of pharmacotherapy on the risk of developing adrenal gland dysfunction (Table 3) in ADHD patients.

Using three models (MPH only, ATX only, and MPH+ATX), we found that neither MPH (aOR, 1.11; 95% CI, 0.79–1.56) nor ATX (aOR, 0.64; 95% CI, 0.23–1.73) significantly influenced the risk of developing adrenal gland dysfunction. Among ADHD patients, an older age at ADHD diagnosis (aOR, 1.14; 95% CI, 1.10–1.17), being female (aOR, 1.79; 95% CI, 1.31–2.46), and having an intellectual disability (aOR, 1.54; 95% CI, 1.07–2.23) were all associated with a greater risk of adrenal gland dysfunction. In addition, we found that duration of MPH/ATX in use and the daily doses did not affect the risk of developing disorders of adrenal glands (Supplementary Table S2).

## 3. Discussion

The main results of our study show that the ADHD group had higher comorbidity rates of adrenal gland disorder compared to the control group. Furthermore, among ADHD patients, an older age at ADHD diagnosis, being female, and having an intellectual disability were all associated with a greater risk of adrenal gland dysfunction. Regarding the relationship between the pharmacotherapy of ADHD and adrenal gland disorder, we found that neither MPH nor ATX significantly influenced the risk of developing adrenal gland dysfunction.

With regard to the comorbidity rates of ADHD and adrenal gland dysfunction, our results showed a one-fold higher rate in the ADHD group than in the control group (0.2% of ADHD vs. 0.1% of controls). To determine whether this higher rate has clinical significance, we had to recognize the prevalence of adrenal gland disorder in the normal population. A common adrenal gland disorder, adrenal insufficiency, can be classified into primary or secondary (or central) adrenal insufficiency based on its etiology. Primary AI is rare, with a prevalence of approximately 93–140 per 1,000,000 (0.0093–0.014%), and is most commonly caused by congenital adrenal hyperplasia (CAH) in children [25]. Secondary AI, which has an intracranial pathology, is also rare, while iatrogenic tertiary AI has an estimated prevalence of 150–280 per 1,000,000 (0.015–0.028%) [15]. The total prevalence of adrenal insufficiency in the normal population is approximately 0.042%, which is much lower than that of the ADHD group (0.2%) in our study, and is also lower than in our control samples (0.1%). Notably, the prevalence of adrenal gland dysfunction in our study was defined using an ICD-9-CM code 255.X, which contained a heterogeneous group of disorders of adrenal glands. Therefore, this finding indicates that ADHD patients have a higher comorbidity rate of adrenal gland disorder than in the normal population. The comparison of the specific adrenal gland disorder between ADHD patients and controls needs further investigation.

The pathogenesis of the comorbidity of ADHD and adrenal gland disorder has yet to be determined, but abnormalities in the HPA axis function may be involved. The HPA axis and resulting glucocorticoid levels have been linked to several cognitive functions, including focusing attention, suppressing unwanted impulses and thoughts, and exerting voluntary behavior [26,27]. Low cortisol levels, especially in challenging situations, may cause behavioral disinhibition and result in the maladaptive behavior of ADHD children.

Possible explanations for the involvement of HPA axis hypoactivity in ADHD children include the following [28]. First, the relatively low cortisol level may be due to chronic stress on the HPA axis. Children with ADHD have higher exposure to adverse childhood experiences compared to children without ADHD [29], resulting in a greater exposure to chronic stress. Another study [30] also reported that boys with prominent ADHD symptoms displayed long-term hypoactivity of the HPA axis, indicated by hair cortisol concentration. Meanwhile, a dysregulation of the HPA axis in ADHD patients due to an underlying dysfunction of the suprachiasmatic nucleus (SCN) in the ventral hypothalamus, regulating circadian variations of psychological, behavioral, and physiologic functions, has been hypothesized [31].

Factors such as being female and having an intellectual disability were found to be associated with a higher risk of adrenal gland dysfunction among ADHD patient in our study. While primary and secondary adrenal insufficiency are both known to occur more frequently in women than in men [15], compared to the control group, the ADHD group in our study had higher comorbidity rates of ODD (5.8%), conduct disorder (6.1%), tic disorders (6.5%), ASD (8.7%), and intellectual disability (14.3%). Literature regarding the comorbidities of adrenal gland dysfunction and intellectual disability is scarce, and this comorbidity has only been observed in children with X-linked adrenoleukodystrophy [32].

Regarding the relationship between the pharmacotherapy of ADHD and adrenal gland dysfunction, neither MPH nor ATX significantly influenced the risk of developing adrenal gland dysfunction in our study. Some studies have previously investigated the effects of MPH or ATX administration on cortisol levels. The salivary cortisol level increased after 60 mg atomoxetine use [20], while cortisol levels would increase at bedtime after using atomoxetine [11]. Significantly increased levels of salivary cortisol were observed after 4-week MPH treatment before decreasing to an intermediate level [17]. However, no previous studies have claimed an association or causal effect between MPH or ATX use and adrenal gland dysfunction. Furthermore, among the causes of adrenal gland dysfunction, several drugs may be accountable for the cause of adrenal insufficiency, including anesthetic-sedative [33] and antimycotic agents [34]. However, no current evidence has revealed that MPH or ATX influences long-term adrenal function.

This study has a number of limitations that should be mentioned at this point. First of all, the inspection of adrenal gland dysfunction was made according to ICD records, so this study lacks relevant laboratory data (e.g., ACTH or cortisol). Therefore, we were unable to validate the diagnostic accuracy of adrenal gland dysfunction using laboratory data. Second, this study is based on reimbursement data from NHIRD-TW, so the diagnosis of ADHD was identified according to ICD records, instead of validated using structural diagnostic instruments. As a result, the classification of ADHD may not be rigorous enough. Third, the database did not show the severity of the symptoms of the affected individuals, and clinically severe patients are more likely to receive pharmacotherapy. Fourth, patients with adrenal gland disorder (ICD-9-CM 255.X) included in our study may consist of heterogenous disorders. While some of these conditions are related to a low cortisol level, others are caused by excessive corticosteroid, which may be less relevant to ADHD. Furthermore, literature regarding all adrenal gland disorders is scarcer than those focusing on specific diseases like adrenal insufficiency or Cushing’s syndrome, making it more difficult to obtain accurate epidemiology data of adrenal gland disorders. Moreover, ATX served as a second-line treatment choice of ADHD prior to 2017. Patients who received ATX treatment may have special characteristics (e.g., poor response to MPH treatment, comorbidity of tic disorders or anxiety disorders). Therefore, the study’s results may be influenced by selection bias. Finally, despite applying a propensity score matching strategy, significant differences in age and sex were still found among the ADHD group and the control group. This matter was the result of the recruited number being so large, so the LHID 2000 cohort could not provide enough perfectly matched controls to the given criteria.

## 4. Conclusions

Patients with ADHD had greater comorbid rates with adrenal gland dysfunction than the control subjects. Nevertheless, receiving pharmacotherapy (MPH or ATX) did not significantly influence the risk of developing adrenal gland dysfunction among ADHD patients. Therefore, continuous efforts are needed to promote public awareness of potential adrenal gland dysfunction among ADHD patients. Furthermore, no current evidence has revealed that MPH or ATX influences long-term adrenal function.

## Figures and Tables

**Table 1:** Characteristics of patients with ADHD and control subjects in Taiwan from 2002 to 2011.

Characteristics	ADHD (*n* = 75,247)	Controls (*n* = 75,247)	Statistics	*p*-Value
Age at diagnosis or recruitment (years)	9.8 ± 4.0	10.2 ± 4.2	20.39	<0.001 *
Gender			2081.39	<0.001 *
Female	15,501 (20.6)	23,239 (30.9)		
Male	59,746 (79.4)	52,008 (69.1)		
Comorbidity				
Oppositional defiant disorder	4360 (5.8)	28 (0.0)	4405.16	<0.001 *
Conduct disorder	4555 (6.1)	167 (0.2)	4209.71	<0.001 *
Tic disorders	4905 (6.5)	609 (0.8)	3474.34	<0.001 *
Autism spectrum disorder	6573 (8.7)	193 (0.3)	6299.23	<0.001 *
Intellectual disability	10,724 (14.3)	699 (0.9)	9520.75	<0.001 *
Received pharmacotherapy	53,674 (71.3)	-	-	-
Methylphenidate	53,407 (71.0)	-	-	-
Age at prescription (years)	10.5 ± 3.6	-	-	-
Duration in use (days)	284.1 ± 365.4	-	-	-
Daily dose (mg)	20.8 ± 10.5	-	-	-
Atomoxetine	3142 (4.2)	-	-	-
Age at prescription (years)	13.0 ± 2.7	-	-	-
Duration in use (days)	149.9 ± 179.7	-	-	-
Daily dose (mg)	35.0 ± 13.3	-	-	-
Diagnosed adrenal gland dysfunction	179 (0.2)	80 (0.1)	37.91	<0.001 *
Age at diagnosis (years)	12.4 ± 6.5	12.5 ± 5.9	0.03	0.976

Note: Data are expressed by *n* (%) or mean ± *SD*; statistic values were expressed using Pearson’s χ^2^ or *t* using an independent *t*-test; ADHD—attention-deficit hyperactivity disorder; *—*p* < 0.05.

**Table 2:** Cox’s proportional models for the risk of diagnosis with adrenal gland dysfunction among youths in Taiwan.

Variables	Unadjusted Model		Adjusted Model	
	HR (95% CI)	*p*-Value	aHR (95% CI)	*p*-Value
ADHD	2.47 (1.73–3.52)	<0.001 *	2.40 (1.64–3.50)	<0.001 *
Age at recruitment	1.14 (1.10–1.18)	<0.001 *	1.15 (1.11–1.19)	<0.001 *
Gender (female vs. male)	1.35 (0.95–1.91)	0.096	1.67 (1.18–2.38)	0.004 *
ODD	1.52 (0.67–2.43)	0.319	1.18 (0.52–2.72)	0.692
Conduct disorder	2.07 (1.09–3.93)	0.027 *	1.21 (0.63–2.35)	0.567
Tic disorders	1.65 (0.84–3.24)	0.144	1.37 (0.69–2.72)	0.365
ASD	1.96 (1.15–3.35)	0.014 *	1.47 (0.83–2.61)	0.184
Intellectual disability	1.79 (1.13–2.81)	0.012 *	1.08 (0.66–1.76)	0.756

Note: ADHD—attention-deficit hyperactivity disorder; ODD—oppositional defiant disorder; ASD—autism spectrum disorder; aOR—adjusted odds ratios; 95% CI—95% confidence interval. *—*p* < 0.05.

**Table 3:** Relationships of pharmacotherapy and diagnoses of adrenal gland dysfunction among patients with ADHD, controlling for sex, age, and psychiatric comorbidities.

	Model 1		Model 2		Model 3	
Variables	aOR (95% CI)	*p*-Value	aOR (95% CI)	*p*-Value	aOR (95% CI)	*p*-Value
Age at ADHD diagnosis	1.14 (1.10–1.17)	<0.001 *	1.14 (1.10–1.17)	<0.001 *	1.14 (1.10–1.17)	<0.001 *
Gender (female vs. male)	1.79 (1.31–2.46)	<0.001 *	1.79 (1.30–2.45)	<0.001 *	1.79 (1.31–2.46)	<0.001 *
ODD	1.15 (0.60–2.19)	0.681	1.19 (0.62–2.27)	0.603	1.16 (0.61–2.23)	0.647
Conduct disorder	1.13 (0.65–1.96)	0.673	1.14 (0.66–1.98)	0.641	1.13 (0.65–1.97)	0.658
Tic disorders	1.00 (0.53–1.90)	0.996	1.02 (0.54–1.95)	0.942	1.03 (0.54–1.95)	0.937
ASD	1.26 (0.78–2.06)	0.346	1.28 (0.78–2.08)	0.328	1.27 (0.78–2.07)	0.331
Intellectual disability	1.55 (1.07–2.23)	0.020 *	1.55 (1.07–2.23)	0.020 *	1.54 (1.07–2.23)	0.021 *
Methylphenidate use	1.10 (0.79–1.54)	0.583	-	-	1.11 (0.79–1.56)	0.540
Atomoxetine use	-	-	0.65 (0.24–1.76)	0.397	0.64 (0.23–1.73)	0.377

Note: ADHD—attention-deficit hyperactivity disorder; ODD—oppositional defiant disorder; ASD—autism spectrum disorder; aHR—adjusted hazards ratios; 95% CI—95% confidence interval. *—*p* < 0.05.

## Supplementary Materials

The following are available online at https://www.mdpi.com/1660-4601/17/10/3709/s1, Table S1: Distribution of disorders of adrenal glands among patients with ADHD and control subjects, Table S2: Relationships of duration and doses of pharmacotherapy and risk of adrenal gland dysfunction among patients with ADHD.
