# Supplementary material for: Attention-Deficit/Hyperactivity Disorder, Its Pharmacotherapy, and Adrenal Gland Dysfunction: A Nationwide Population-Based Study in Taiwan
Source: Int J Environ Res Public Health. 2020 May 25;17(10):3709. doi: 10.3390/ijerph17103709 (PMC7277140; doi:10.3390/ijerph17103709)
Supplement: Supplementary file 1 [file ijerph-17-03709-s001.pdf]

Supplementary Table S1. Distribution of disorders of adrenal glands among patients with ADHD and control subjects.

255 Disorders of adrenal glands	ADHD (N = 75247)	Controls (N = 75247)	Statistics	*P*-value
255.0 Cushing's syndrome	72 (0.1%)	31 (<0.1%)	16.33	<0.001*
255.1 Hyperaldosteronism	5 (<0.1%)	7 (<0.1%)	0.33	0.774
255.2 Adrenogenital disorders	36 (<0.1%)	10 (<0.1%)	14.70	<0.001*
255.3 Other corticoadrenal overactivity	4 (<0.1%)	5 (<0.1%)	0.11	1.000
255.4 Corticoadrenal insufficiency	44 (0.1)	17 (<0.1%)	11.96	0.001*
255.5 Other adrenal hypofunction	6 (<0.1%)	2 (<0.1%)	2.00	0.180
255.6 Medulloadrenal hyperfunction	0 (0%)	0 (0%)	-	-
255.8 Other specified disorders of adrenal glands	10 (<0.1%)	8 (<0.1%)	0.22	0.637
255.9 Unspecified disorder of adrenal glands	22 (<0.1%)	7 (<0.1%)	7.76	0.005*

Data is expressed by n (%); Statistic values were expressed using Pearson's χ^2; ADHD, attention-deficit hyperactivity disorder; *P<0.05

Supplementary Table S2. Relationships of duration and doses of pharmacotherapy and risk of adrenal gland dysfunction among patients with ADHD.

	aOR (95% CI)	p-value
Duration of methylphenidate use	1.00 (0.99-1.00)	0.470
Methylphenidate daily doses	1.04 (0.95-1.14)	0.361
Duration of atomoxetine use	0.99 (0.98-1.01)	0.485
Atomoxetine daily doses	0.98 (0.89-1.08)	0.691
